# Stage IV Colorectal Cancer Management and Treatment

**DOI:** 10.3390/jcm12052072

**Published:** 2023-03-06

**Authors:** Oscar Hernandez Dominguez, Sumeyye Yilmaz, Scott R. Steele

**Affiliations:** Department of Colorectal Surgery, Digestive Disease and Surgery Institute, Cleveland Clinic, Cleveland, OH 44195, USA

**Keywords:** metastatic colorectal cancer, stage IV colon cancer, stage IV rectal cancer, treatment of stage IV colorectal cancer

## Abstract

(1) Background: Colorectal cancer (CRC) is the third most common cancer and the second leading cause of cancer-related mortality worldwide. Up to 50% of patients with CRC develop metastatic CRC (mCRC). Surgical and systemic therapy advances can now offer significant survival advantages. Understanding the evolving treatment options is essential for decreasing mCRC mortality. We aim to summarize current evidence and guidelines regarding the management of mCRC to provide utility when making a treatment plan for the heterogenous spectrum of mCRC. (2) Methods: A comprehensive literature search of PubMed and current guidelines written by major cancer and surgical societies were reviewed. The references of the included studies were screened to identify additional studies that were incorporated as appropriate. (3) Results: The standard of care for mCRC primarily consists of surgical resection and systemic therapy. Complete resection of liver, lung, and peritoneal metastases is associated with better disease control and survival. Systemic therapy now includes chemotherapy, targeted therapy, and immunotherapy options that can be tailored by molecular profiling. Differences between colon and rectal metastasis management exist between major guidelines. (4) Conclusions: With the advances in surgical and systemic therapy, as well as a better understanding of tumor biology and the importance of molecular profiling, more patients can anticipate prolonged survival. We provide a summary of available evidence for the management of mCRC, highlighting the similarities and presenting the difference in available literature. Ultimately, a multidisciplinary evaluation of patients with mCRC is crucial to selecting the appropriate pathway.

## 1. Introduction

Colorectal cancer (CRC) is the third most common cancer and the second leading cause of cancer-related mortality worldwide, with an estimated 1.9 million cases and 935,000 deaths annually [1,2]. In the United States, close to 1.37 million people were living with CRC and it is estimated that there were 52,580 deaths in 2022, making CRC the second most common cause of cancer-related deaths [3,4]. The average 5-year relative survival rate for all stages of CRC is 65.1% [3]. However, the cancer stage has a strong influence on survival. In stage IV CRC (metastatic CRC (mCRC)), defined as cancer spread to distant sites or organs or peritoneal metastasis, the 5-year survival rate significantly drops to 15.1% [3].

Approximately 22% of CRC cases have metastasis at presentation, and 19% will develop metachronous metastasis [3,4,5,6]. The most common sites of metastasis listed in order are the liver, lung, and peritoneum [5,7]. The site of metastasis has an impact on survival. To reflect this, stage IV mCRC is further classified based on metastasis to one site or organ (IVa), multiple sites or organs (IVb), or whether peritoneal metastasis is present (IVc). In addition to the site of metastasis, studies have also shown that the timing, number, and originating location of metastasis may also impact survival [6,7,8]. The heterogeneity of mCRC has demanded continued development in diagnosis and pretreatment testing as this is crucial in facilitating a multi-disciplinary approach to treatment.

The standard of care for mCRC primarily consists of surgical resection and systemic therapy. Unlike other stage IV cancers, surgical resection of mCRC can significantly prolong survival. In some cases, resection of liver and lung mCRC can even be curative [9,10,11]. A survival advantage has even been shown in peritoneal metastasis versus palliative care [12,13]. Additionally, the evolution of systemic therapy has improved mCRC survival or allowed the conversion of unresectable mCRC to resectable [14]. More recent additions of treatments targeted therapy of specific genetic tumor mutations (e.g., EGFR, VEGF, KRAS) and immunotherapy agents have also provided survival benefits in certain mCRC [9,11,15,16,17,18]. As the spectrum of treatment options for mCRC has grown, the cost of treating mCRC has also widened. Studies report the average costs of treatment can range from $12,000 to almost $300,000 due to the heterogeneity of treatment plans [19].

As research continues to uncover tumor-specific treatment options in mCRC, understanding the variety of treatment options is essential for increasing patient survival and decreasing the healthcare burden. This review summarizes current evidence regarding the management of mCRC to provide critical aid when making a treatment plan for the heterogenous spectrum of mCRC.

## 2. Materials and Methods

A comprehensive literature search of the Cochrane Database of Collected Research, PubMed, and EMBASE was performed. Our search strategy included different combinations of terms related to ‘stage IV CRC’, ‘colorectal metastases’, ‘diagnosis of stage IV CRC’, ‘surgery for mCRC’, ‘systemic treatment for mCRC’ in “All fields”, and the related Mesh terms to identify English-language publications. Current guidelines written by major cancer societies, including the National Comprehensive Cancer Network (NCCN) [20,21], the European Society of Medical Oncology (ESMO) [22], the Japanese Society for Cancer of the Colon and Rectum (JSCCR) [23], the American Society of Colon and Rectal Surgeons (ASCRS) [24] are also reviewed. The references of the included studies were screened to identify additional studies that were incorporated as appropriate.

We did not seek Institutional Review Board approval as this type of study, a literature review does not require approval since no patient data are accessed or analyzed.

## 3. Diagnosis of Stage IV Colorectal Cancer

Staging has critical implications for the treatment plan and survivability of patients with mCRC. Diagnostic staging of mCRC mainly consists of laboratory tests and imaging, with a similar foundation as CRC staging. Adjunct imaging is mostly required to determine if a patient with mCRC can undergo curative resection.

For suspected or proven synchronous mCRC, guidelines suggest a total colonoscopy with biopsy be completed. Laboratory tests should include a complete blood count (CBC), a chemistry panel, and baseline carcinoembryonic antigen (CEA). In recent studies, elevated carbohydrate antigen (CA) 19-9 levels have been reported to be a predictor of poor survival [25,26]. Nevertheless, obtaining a CA 19-9 is optional per ESMO guidelines and not required by NCCN guidelines [20,21,22]. A computed tomography (CT) with intravenous (IV) and oral contrast of the chest, abdomen, and pelvis has long been the staple imaging modality for CRC [27]. Magnetic resonance imaging (MRI) with IV contrast of the abdomen can be helpful in cases where CT is insufficient in evaluating a metastatic lesion, particularly for the operative evaluation of metastatic liver lesions [28]. A positron emission tomography (PET)/CT scan is not recommended in routine diagnosis, staging, or surveillance. PET/CT should mainly be reserved for select cases of potentially resectable mCRC lesions. For example, during the preoperative evaluation of patients with high suspicion of previously unrecognized, or high extent mCRC that would exclude surgery [20,22]. The same laboratory testing and imaging recommendations apply to metachronous mCRC.

Subtle, but essential differences are present between colon and rectal cancer diagnostic staging. For the evaluation of rectal cancer, a pelvic MRI with contrast is a crucial addition for diagnosis, treatment, and surveillance due to its superior capability of evaluating tumor depth and prediction of circumferential resection margin (CRM) [21,29,30,31]. Furthermore, MRI detection of involved CRM has been reported to have a significant association with distant metastatic disease [30]. Endoscopic ultrasound (EUS) can also be used to evaluate rectal cancer. However, due to its decreased accuracy in staging and operator dependence, recent guidelines recommend EUS be reserved mainly when MRI is contraindicated [32,33]. A proctoscopy can be considered for the evaluation of rectal cancer. Proctoscopy can help determine an accurate distance between the anal verge and the primary tumor, which is important when determining if it is a low colon versus rectal cancer [34].

## 4. Treatment Strategies for Stage IV Colorectal Cancer

Treatment of mCRC is challenging and involves different modalities such as chemotherapy, radiotherapy (RT), and surgery. Multidisciplinary evaluation of patients is crucial, as there is no absolute treatment strategy [24]. Treatment strategies and goals are determined according to:

Tumor- and disease-related factors (e.g., synchronous or metachronous metastases, location and resectability of metastases and the primary tumor, presence of primary tumor-related symptoms, pathology, and molecular profiling).

Patient-related factors (e.g., Eastern Cooperative Oncology Group (ECOG) performance status [35], co-morbidities, patient expectations).

Treatment-related factors (e.g., toxicity) [20,21,22,23].

### 4.1. Treatment of Synchronous Metastases

**Figure 1:**
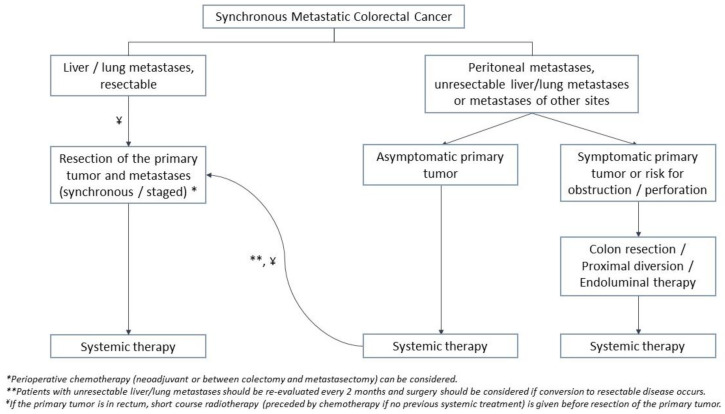
Treatment Algorithm for Metastatic Synchronous Adenocarcinoma of the Colon and Rectum.

Treatment of synchronous mCRC largely depends on the location and resectability of metastases. As a general rule, surgical resection combined with systemic treatment achieves the best cure in patients with resectable metastases. Whereas in the setting of unresectable disease, management depends on the degree of primary tumor-related symptoms. The treatment algorithm based on NCCN [20,21] and ASCRS [24] guidelines is summarized in Figure 1.

#### 4.1.1. Management of the Primary Tumor in the Setting of Unresectable Disease

The most common symptoms associated with CRC are bleeding, obstruction, and perforation. Chronic bleeding oftentimes presents itself with anemia, which can be managed non-operatively. However, acute significant blood loss requires intervention. For proximal cancers, resection is preferred [24]. Other treatments such as endovascular procedures (e.g., embolization, coiling, stents) [36,37] and radiation [38] can provide quick and effective palliation, with the latter being particularly effective in the rectum.

About 8–30% of patients with CRC present with partial or complete obstruction [24]. Further, up to 6% of patients with unresectable mCRC eventually require urgent surgical treatment due to obstruction or perforation [39], which is associated with reduced overall survival (OS) [24,40]. Compared to surgery, treating malignant obstruction with a colonic stent is associated with faster recovery, fewer complications, and a shorter time to chemotherapy onset, which is an important predictor of OS [41,42,43]. However, distal rectal tumors may not be amenable to stenting. Primary tumors that cause bowel perforation are associated with a poor prognosis [44], and resection is the preferred treatment.

Resection of the primary asymptomatic tumor in the presence of unresectable mCRC is not recommended [20,21]. In their review, Cirocchi et al. [39] failed to show an association between primary tumor resection with OS. Kanemitsu et al. [45] published a randomized controlled trial (RCT) showing no survival benefit of primary tumor resection in the setting of unresectable mCRC. It seems like these patients die as a result of their systemic disease rather than primary tumor-related complications [39].

#### 4.1.2. Liver Metastases

The liver is the most common metastatic site for mCRC. Around 11.8–14.4% of patients with colon and 9.5–12.5% with rectum cancer has liver metastases at the time of diagnosis [23,46]. The most common route of dissemination is hematogenous via the portal system [47].

According to European Colorectal Metastases Treatment Group (ECMTG), colorectal liver metastases (CRLMs) are classified into four groups: M0 (no metastases), M1a (resectable metastases), M1b (potentially resectable liver metastases), and M1c (liver metastases that are unlikely to ever become resectable) [48]. At the time of diagnosis, 20–25% of patients with CRLMs have resectable or potentially resectable lesions [24,49]. Surgery represents the only curative option for resectable CRLM. Current guidelines recommend performing a CRLM resection when curative resection (R0—resection with microscopically negative margins) is possible [20,21,22,23,24], there are no uncontrollable extrahepatic metastases, the function of the remaining liver is adequate, and the patient is capable of tolerating surgery [20,23]. Alternative to upfront resection, perioperative chemotherapy can be administered, which may increase progression-free survival (PFS) and OS [50,51]. Five-year OS rates following surgery are above 30% [52,53], approaching 58% in selected patients [23,54,55].

In the setting of unresectable synchronous liver metastases, systemic chemotherapy is recommended, with surgery being reserved for patients with primary tumor-related symptoms [20,22,24]. Re-evaluation should be performed every two months [20,22], as conversion from unresectable to resectable disease can be observed in 15–50% of patients [47]. Following conversion chemotherapy, R0 resection of hepatic metastases is associated with better survival than if resection was not performed [22,56]. Therefore, synchronous or staged resection of the primary tumor and metastases is recommended if conversion to resectable disease occurs [20,24]. The treatment algorithm and goals for colorectal liver or lung metastases are summarized in Figure 1 and Table 1.

Ideally, R0 resection of CRLMs should be performed while maintaining adequate hepatic function. To maintain adequate liver function and prevent post-hepatectomy liver failure, future liver remnant (FLR) should be more than 20% of the total liver volume in a healthy, chemotherapy-naïve patient, 30% in a chemotherapy-treated patient, and 40% in patients with any evidence of cirrhosis or fibrosis [9,57]. Increasing emphasis on preserving the volume of FLR has led to several treatment strategies. Parenchymal-sparing hepatectomy (PSH), one of these strategies, is a widely-practiced surgical approach aiming to achieve R0 resection of the tumor while maintaining as much liver parenchyma as possible. Deng et al. [58] conducted a meta-analysis comparing the outcomes of PSH vs. non-PSH and reported comparable 3-year and 5-year OS and recurrence-free survival (RFS) rates. Furthermore, non-PSH was associated with higher postoperative complications and 90-day mortality rates. In their systematic review, Moris et al. [59] also reported comparable OS rates for PSH vs. anatomic resection. Nevertheless, the decision to proceed with PSH or non-PSH is made based on multiple factors such as tumor number, size, location [60,61], and the presence of specific mutations [62]. There is no clear consensus regarding the resection margin during PSH [20,24,63].

Regarding the timing of the surgery, simultaneous or staged (metachronous) resection of the primary tumor and metastases can be performed. Traditionally, resection of the primary tumor followed by systemic chemotherapy and resection of metastases (bowel-first) has been preferred. The liver-first strategy was proposed to address metastatic disease first, as it is the primary determinant of OS [64]. Depending on the difficulty of the colectomy or hepatectomy, the medical condition of the patient, and the surgeon’s expertise, simultaneous colon and liver resection can safely be performed without compromising long-term oncological outcomes [65,66,67]. However, simultaneous resection is avoided when there is a primary tumor-related complication such as bleeding, obstruction, and perforation [52].

Dealing with a higher volume of total liver tumors or multiple bilobar liver disease requires more advanced strategies, as extended hepatectomy might result in post-hepatectomy liver failure [9]. When hepatic metastases are considered unresectable due to inadequate FLR, two-stage hepatectomy with or without portal vein embolization (PVE) can be considered [68]. This technique involves complete resection of metastases from the FLR (first stage hepatectomy), followed by PVE 2–5 weeks after the first stage hepatectomy, and second stage hepatectomy [69,70]. Dropout after the first stage is the major drawback of this operation, which can be seen in up to 35% of the patients [71], majorly due to tumor progression and sometimes insufficient FLR volume. Nevertheless, a 5-year survival rate of up to 64% for patients who complete the second stage reinforces the benefit of this procedure [72].

Associating liver partition and portal vein ligation for staged hepatectomy (ALPPS) involves portal vein ligation with tumoral clearance of the FLR and in situ splitting of the liver parenchyma, followed by a second operation 1–2 weeks later [70]. Compared to other techniques, ALPPS is associated with the highest growth rate for the FLR [73,74]. With ALPPS, more patients complete the second stage of hepatectomy, and the waiting interval between the two stages is shorter [74,75,76,77,78]. Although some RCTs and meta-analyses reported similar outcomes [75,76], the major concern about ALPPS remains to be the high postoperative morbidity and mortality rates [74,78]. Nevertheless, OS and RFS rates seem to be similar for two-stage hepatectomy and ALPPS [77,78].

Local ablative treatments (e.g., radiofrequency ablation (RFA), microwave ablation (MWA)) are commonly used in the treatment of CRLMs to increase resectability and achieve curative treatment in poor surgical candidates and patients with unresectable disease due to unfavorable tumor location or multilobar metastases. When combined with systemic therapy, RFA and MWA offer better disease-free survival and OS compared to chemotherapy alone [79,80,81]. These techniques may also be safely used as an adjunct to surgery in the presence of CRLMs, providing RFS and OS rates similar to surgery alone [82]. Transarterial chemoembolization (TACE) and radioembolization (TARE) are considered salvage therapies for patients with CRLMs not amenable to surgery or ablation who fail systemic chemotherapy [24]. Stereotactic body radiation therapy (SBRT) delivers high-dose radiation to tumoral tissues. It may be considered for the treatment of unresectable CRLMs and in the setting of oligometastatic disease (OMD), however, limited data exists on the outcomes of SBRT for CRLMs [83,84].

Liver transplantation has previously been proposed as a treatment option for unresectable CRLMs [85]. However, due to the high recurrence rate and poor OS, liver transplantation for CRLMs was abandoned. Groundbreaking research from Oslo [86] reintroduced liver transplantation for CRLMs, with 5-year OS rates reaching 60%. Predictors of survival in the first study led authors to define the Oslo Score (Table 2) and design the second study (SECA-II), which showed that with more strict selection criteria, 5-year OS rates of 83% can be achieved [87]. Although limited by organ availability, liver transplantation may offer a promising treatment option for patients with unresectable CRLMs.

#### 4.1.3. Lung Metastases

Lungs are the second most common site for mCRC after the liver. Approximately 10–22% of patients with CRC have lung metastases at the time of diagnosis [24,52,88]. While cancers of the colon and upper rectum drain into the liver via the portal system, cancers of the middle and lower rectum can metastasize directly to the lungs via the inferior hemorrhoidal vein and inferior vena cava [89]. Due to this anatomical difference, pulmonary metastases are seen more frequently in rectal cancer than in colon cancer [52,89]. Pulmonary metastases have slower growth and better survival than other metastases [90]. Following pulmonary metastasectomy, 5-year OS is usually above 50%, reaching up to 68% in patients with isolated lung metastases [24,52,89,90,91].

Management of lung metastases is similar to CRLMs (Figure 1 and Table 1) with surgical resection being the preferred practice in the setting of resectable disease [20,24,92]. However, unlike CRLMs, the literature on the outcomes of pulmonary metastasectomy is limited to retrospective case series, without a single prospective RCT [24,92]. The goal is to achieve R0 resection with the preservation of adequate pulmonary function. Usually, the tumor is amenable to wedge resection or segmental resection. A resection margin of 10 mm is recommended by several studies [93]. Studies reported no difference in RFS and OS after video-assisted thoracoscopic surgery (VATS) vs. open thoracotomy. However, lack of intraoperative palpation during VATS may preclude the detection of small metastases [94]. Pulmonary lobectomy in the setting of metastatic disease may be associated with poor prognosis [90]; therefore, is not recommended [93].

Mediastinal and hilar lymph node positivity can be seen in up to 44% of patients with pulmonary metastases, and it is associated with poor prognosis [89,90,95]. Dissection of the mediastinal and hilar lymph nodes can be considered; however, this has not improved survival [90].

Local ablative treatments can be used either alone or in conjunction with surgery for resectable pulmonary metastases. They can also be considered for OMD, unresectable metastases, or patients with high operative risk [20,24]. When surgical resection is not feasible, RFA and MWA are associated with improved survival [96]. For tumors larger than 3 cm, MWA and SBRT seem to be more effective in achieving local tumor control [97]. Data comparing SBRT to other techniques is scarce, nevertheless, it can be considered in highly selected cases such as centrally located lesions in the setting of OMD [97].

#### 4.1.4. Peritoneal Metastases

Peritoneal metastases are seen in 5–17% of mCRC patients [9,52,98,99]. Underlying mechanisms include spontaneous seeding of tumor cells from a T4 CRC, extravasation of tumor cells as a result of either spontaneous or iatrogenic perforation, and transection of lymphatics during colon resection [100]. The most frequent signs and symptoms are ascites and intestinal obstruction [99]. Peritoneal metastasis is a well-known predictor of poor prognosis in CRC patients [101]. In fact, until recent advances in therapy, the presence of peritoneal metastases was thought to be representing a terminal disease [9].

According to NCCN guidelines, the primary treatment of peritoneal metastases is systemic chemotherapy. Surgical resection of the primary tumor should be considered in the presence of primary tumor-related symptoms, or imminent risk for obstruction (Figure 1) [20].

More recently, cytoreductive surgery (CRS) with or without hyperthermic intraperitoneal chemotherapy (HIPEC) has been performed for the treatment of CRC peritoneal metastases. CRS involves the removal of all macroscopic tumor tissue, involving peritonectomy, resection of involved organs, and omentectomy. HIPEC comprises intraperitoneal circulation of chemotherapeutics (most commonly oxaliplatin or mitomycin-C (MMC) based) to eradicate microscopically remnant tumor cells [52,99]. In their RCT, Verwaal et al. [102] reported increased OS for patients undergoing CRS + HIPEC (MMC-based) + adjuvant therapy vs. systemic therapy alone. Their study was limited by the inclusion of appendiceal carcinomas and patients discontinuing treatment due to toxicity or disease progression. Cashin et al. [103] designed an RCT comparing outcomes for patients undergoing CRS + HIPEC vs. systemic chemotherapy. Although terminated prematurely, their study managed to prove increased OS for patients undergoing CRS + HIPEC. However, it seems the completeness of cytoreduction is the predictor of OS [102,103,104]. PRODIGE 7 RCT [105] evaluated the role of HIPEC by comparing CRS + HIPEC vs. CRS alone. Their results showed no difference in OS. Additionally, Grade 3+ adverse events at 60 days were higher in the CRS + HIPEC group. Today, several guidelines recommend CRS plus chemotherapy for selected patients (Table 3) with peritoneal metastases [9,99,106]. However, HIPEC is not routinely recommended as an addition to CRS [22,83].

Synchronous colorectal liver and peritoneal metastases (CLPM) can be seen in 8% of stage IV CRC patients [98]. Studies report OS ranging from 13 to 45.7 months with a combination of CRS + HIPEC with liver resection (or ablation) for synchronous CLPM [107,108]. Nevertheless, the best strategy for the treatment of CLPM remains unclear [107].

#### 4.1.5. Other Metastases

Approximately 5–10% of women with CRC develop ovarian metastasis, which is associated with a median OS of 19–27 months [109]. Ovarian metastasis can reach significant sizes without becoming symptomatic. Compared to other sites, ovarian metastasis is disproportionately unresponsive to chemotherapy. Several studies report better survival outcomes with surgical oophorectomy and cytoreduction, especially when R0 resection is achieved [109,110]. Routine prophylactic oophorectomy can be offered to postmenopausal women with mCRC, however, is not recommended for premenopausal women [24]. Recently, a Dutch trial has been proposed, aiming to evaluate the role of prophylactic oophorectomy in postmenopausal women, but no results have been published yet [111].

In addition to ovarian metastasis, CRC can metastasize to several other organs, including bone, brain, adrenal glands, and retroperitoneal lymph nodes. Bone metastasis is seen in 6–10.4% of the cases and is associated with poor OS [112]. Palliation of pain is an important part of treatment in patients with bone metastasis. Brain metastasis is rare (1–4%). Surgical resection of solitary metastasis is associated with OS of 30–40 weeks [23], and a local recurrence rate of 50–60% [24]. Stereotactic radiation therapy can achieve an 80–90% local control rate in patients with no more than 3–4 metastases that are <3 cm in diameter [23,24]. Whole brain radiation therapy (WBRT) can be an option for patients with multiple brain metastases, however, it is associated with increased side effects, and no improvement in OS [113]. Although there are controversies regarding the treatment of retroperitoneal and para-aortic lymph node metastases, a meta-analysis recently has shown improved OS following the resection of para-aortic lymph nodes, with no increase in postoperative complication rates [114].

#### 4.1.6. Rectal Cancer

**Figure 2:**
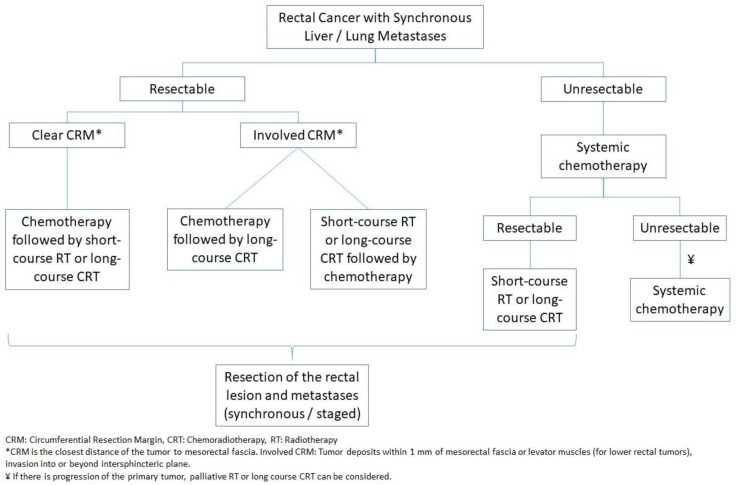
Treatment Algorithm for Stage IV Rectal Cancer with Synchronous Liver/Lung Metastases.

Management of stage IV rectal cancer with synchronous lung and/or liver metastases is different from colon cancer by the addition of RT to the treatment algorithm [21] (Figure 2). RT can be given either as short-course RT (25 Gy in five daily treatment fractions) or long-course chemoradiotherapy (CRT) (45–50.4 Gy in 25–28 fractions over 5–6 weeks with concurrent 5-FU infusion or capecitabine). Perioperative RT combined with surgery results in better OS [45,115]. Furthermore, preoperative RT is preferred over postoperative RT, because it is associated with reduced local recurrence risk [116,117,118]. In some patients, a complete response of the primary tumor to RT can be seen. In these patients, the watch-and-wait (W&W) approach can be considered. However, this approach can be associated with a high local regrowth rate [119]. Moreover, literature regarding the use of the W&W approach in stage IV rectal cancer is very limited.

Surgery can be performed immediately (within 1 week) or delayed (4–6 weeks) after RT. The ideal timing of surgery in relation to RT remains controversial. In the Stockholm III trial [120], patients with stage IV rectal cancer were divided into three groups (short-course RT (SCRT) with immediate surgery, SCRT with delayed surgery, and long-course RT with delayed surgery) to address this question. The results of this trial showed no difference between the three groups in cumulative incidences of local recurrence, distant metastases, and OS. In two-arm randomization, postoperative complications were more common in SCRT with delayed surgery group compared to SCRT with immediate surgery.

Although the timing of surgery is not important according to the Stockholm III trial [120], delaying hepatic resection until completion of systemic chemotherapy is concerning due to chemotherapy-induced liver changes, which may increase the risk for post-hepatectomy liver failure. Therefore, the current ASCRS guideline’s recommendation is to perform immediate surgery following chemotherapy and SCRT if RT is planned after chemotherapy [24]. Another possible option can be to perform hepatic resection after 2–3 months of neoadjuvant chemotherapy, with an additional 3 months of chemotherapy followed by RT and then resection of the primary tumor.

### 4.2. Treatment of Metachronous Metastases

Up to 14–34% of patients with non-metastatic CRC will ultimately develop metachronous metastases [121]. Like synchronous metastases, the liver and lung are the most common site for metachronous metastases. The prognosis of CRC with metachronous metastases is better than CRC with synchronous metastases, with a recent study reporting 49.9% vs. 41.8% 1-year OS, and 13.2% vs. 6.2% 5-year OS in favor of metachronous CRLMs [122].

**Figure 3:**
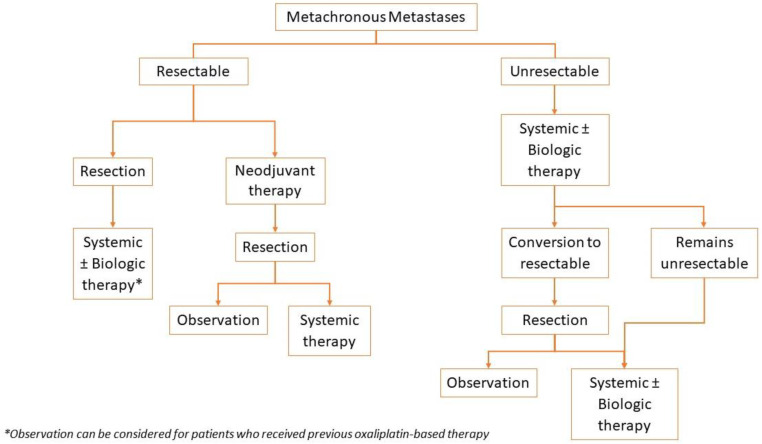
Treatment Algorithm for Metastatic Metachronous Adenocarcinoma of the Colon and Rectum.

The treatment algorithm for CRC with metachronous metastases is summarized in Figure 3. When possible, resection of metastases is preferred. Whereas if the disease is unresectable, systemic therapy is the main treatment. Patients with an unresectable disease should be followed every 2 months for conversion to resectable disease. If conversion to resectable disease occurs, resection followed by observation or systemic therapy is recommended [20,21].

### 4.3. Systemic Therapy

**Figure 4:**
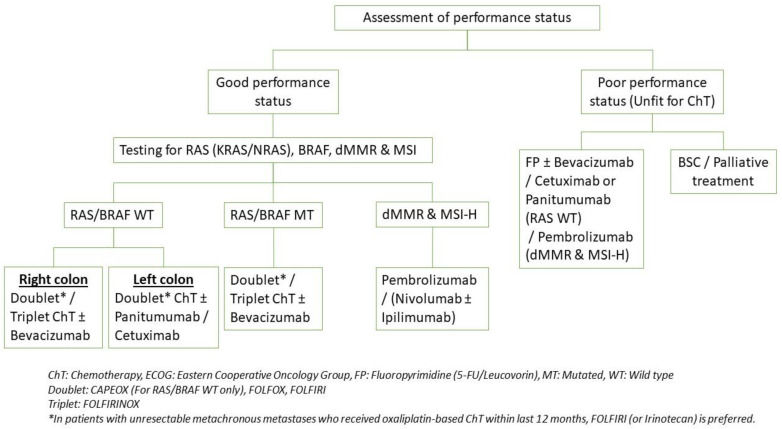
First-line Systemic Treatment for Stage IV CRC with Unresectable Metastases.

**Figure 5:**
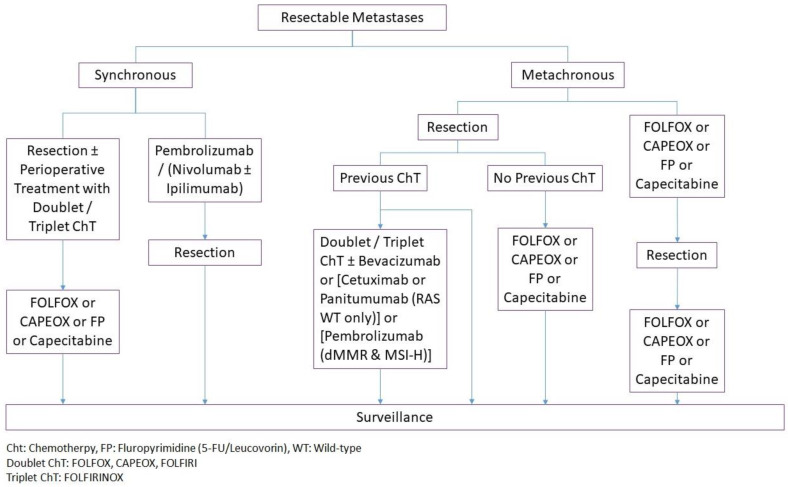
First-line Systemic Treatment for Stage IV CRC with Resectable Metastases.

Along with surgery, systemic therapy is one of the primary components of mCRC treatments (Figure 4 and Figure 5). Systemic therapy can offer a curative option or a palliative treatment. Advances in systemic therapy options and efficacy improved survival from 6 to 12 months to 2 to 3 years [16]. More recently, systemic therapy has played a growing role in unresectable mCRC, where it is increasingly being used to convert patients with an unresectable disease into suitable candidates for resection. Even among patients who initially undergo resection, systemic therapy is used to target occult nodal infiltration and micro-metastasis. Given the constant evolution and high complexity of systemic therapy, all patients with mCRC should be evaluated in a center with multidisciplinary specialists to help them determine the best combination of chemotherapy, targeted therapy, radiation, and available clinical trials.

Chemotherapy is the mainstay of systemic treatment for mCRC. Chemotherapy regimens will typically consist of a fluoropyrimidine (5-FU or capecitabine) paired in a two-drug regimen (doublet) with irinotecan or oxaliplatin. Treatment regimens can be 5-FU- or capecitabine-based and can be either oxaliplatin-based (FOLFOX or CAPEOX) or irinotecan-based (FOLFIRI or CAPIRI) with no difference in survival [123,124,125,126]. With similar efficacy between combinations, toxicity profiles can distinguish the optimal treatment regimen for individual patients. It is not uncommon for patients to transition between chemotherapy combinations due to intolerance of side effects or disease progression.

Bevacizumab, a monoclonal antibody that targets vascular endothelial growth factor (VEGF), has become a part of mCRC treatment starting in 2004 after an RCT showed OS advantages when bevacizumab was paired with 5-FU-based chemotherapy [127,128]. Treatment with fluoropyrimidine and bevacizumab has been shown to provide longer PFS in elderly patients unable to tolerate more aggressive doublet first-line treatments [129,130]. Bevacizumab can also be added to doublet chemotherapy in first-line therapy for select patients [131,132,133]. However, bevacizumab is not recommended in the perioperative period due to the risk of wound healing complications [128]. NCCN guidelines recommend holding bevacizumab 6 weeks before surgery and 6 to 8 weeks post-surgery after several studies found no difference in perioperative complications when bevacizumab was held during this period [20,21,134,135].

Regimens with a three-drug (triplet) combination, FOLFIRINOX or FOLFOXIRI, are also available as first-line therapy and are commonly paired with bevacizumab. Major clinical guidelines recommend triplet therapy in patients that are optimally fit and without significant comorbidities [20,21,22,23]. NCCN recently updated their guidelines to replace the higher 5-FU dose in FOLFOXIRI with a lower 5-FU-dosed FOLFIRINOX due to patients in the USA having greater toxicity with 5-FU [20,21]. A meta-analysis by Cremolini et al. [136] evaluated unresectable mCRC European-based patients randomly assigned to either FOLFOXIRI plus bevacizumab or doublet regimen plus bevacizumab and found a significant benefit in OS and R0 resection rate in the FOLFOXIRI group. Of note, higher rates of neutropenia, mucositis, nausea, and diarrhea were observed and 99% of the patients in this analysis had an ECOG performance status from 0 to 1. However, the randomized phase III TRIPLETE study recently showed that modified FOLFOXIRI failed to provide benefits in overall response rate or R0 resection rates when compared to modified FOLFOX, but significantly increased GI toxicity side effects were seen with triplet therapy [137].

#### 4.3.1. Molecular Profiling

The ability to analyze individual tumors for biomarkers (genes, proteins, or other molecules) has driven the growth of targeted therapy for mCRC. Currently, aberrations in microsatellite instability (MSI), mismatch repair (MMR), KRAS, NRAS, BRAF, and HER2 genes have been identified as significant biomarkers impacting therapy response. As a result, all major guidelines uniformly recommend that mCRC cases undergo molecular profiling at initial diagnosis to help personalize the most favorable systemic therapy regimen [20,21,22,23].

All major guidelines recommend patients with mCRC undergo testing for MMR deficiency (dMMR) or high MSI (MSI-H) [20,21,22,23], a mutation found in up to 5% of mCRC [138]. Universal testing of dMMR/MSI-H can help identify and counsel patients with Lynch syndrome, the most common cause of hereditary CRC, and it can also be a predictive marker for immunotherapy susceptible tumors. Tumors with dMMR/MSI-H evade immune-system destruction by blocking the PD-1 receptors on host T cells. PD-1 inhibitors (e.g., Nivolumab, Pembrolizumab) and cytotoxic T-lymphocyte associated protein 4 (CTLA-4) inhibitors (e.g., Ipilimumab) are immunotherapy treatments aimed at preventing dMMR/MSI-H tumors from escaping destruction. Clinical trials have shown that targeting dMMR/MSI-H mCRC with PD-1 inhibitors and CTLA-4 inhibitors results in a beneficial clinical response and improved survival [139,140]. Based on a recent analysis of the KEYNOTE-177 trial [141], most guidelines now recommend that pembrolizumab alone can be used as first-line therapy in dMMR/MSI-H mCRC.

Molecular profiling can also identify mutations that predict ineffective therapies. For example, mCRC tumors with mutations in the RAS gene (KRAS or NRAS), seen in approximately 40% of mCRC [142], have shown no treatment benefit from epidermal growth factor receptor (EGFR) inhibitors (e.g., panitumumab, cetuximab) [143,144]. Therefore, EGFR inhibitors are not recommended in patients with RAS-mutated mCRC. Moreover, determining RAS status with primary tumor location has been shown to affect treatment response. Wild-type RAS mCRC whose primary tumor originated in the right colon (cecum to hepatic flexure) has shown little benefit when treated with EGFR inhibitors but did show a favorable response with Bevacizumab [145,146,147]. Therefore, current guidelines suggest EGFR inhibitor treatment should only be first-line for wild-type RAS mCRC whose primary tumor originated from the left colon (splenic flexure to rectum) and bevacizumab for right-side originating mCRC [20,21,22,23,148].

BRAF V600E-variant tumors, found in approximately 6–9% of patients with mCRC [138], also do not benefit from treatment with EGFR inhibitors [149,150]. As a result, EGFR inhibitors are no longer included in the first-line treatment of the BRAF variant mCRC. However, longer survival and improved treatment response rates have been observed when the BRAF variant mCRC is treated simultaneously with EGFR inhibitors and BRAF inhibitors (e.g., encorafenib) [151,152]. EGFR inhibitors paired with BRAF inhibitors can be used as second-line treatment for wild-type RAS and BRAF V600E-variant mCRC. Additionally, BRAF mutations are a marker of a more aggressive phenotype and poor prognosis [153]. Multidisciplinary boards should consider the poor prognosis of this variant when determining therapy or inclusion in clinical trials.

Most recent ESMO and NCCN guidelines recommend testing wild-type RAS and wild-type BRAF mCRC patients for human epidermal growth factor receptor 2 (HER2) amplification, which is found in less than 5% of mCRC [20,21,22,154,155]. This recommendation comes as several studies have shown that mCRC tumors without mutations in RAS or BRAF, but exhibiting HER2 amplification, have favorable responses when treated with HER2 antagonists (e.g., trastuzumab, pertuzumab) [156,157]. When feasible, guidelines also recommend testing for NTRK fusions, which are found in less than 1% of CRC [158]. This is supported by multiple studies showing mCRC solid tumors with NTRK fusions had a favorable response rate when treated with NTRK inhibitors (e.g., larotrectinib and entrectinib) [159,160].

#### 4.3.2. Maintenance Therapy

Continuation of induction therapy becomes difficult after achieving maximum response due to neuropathy and fatigue associated with oxaliplatin-based chemotherapy and FOLFIRI, respectively [161]. The concept of maintenance therapy includes reducing treatment intensity without compromising disease control [22]. It is used after first-line therapy for patients with unresectable mCRC until disease progression. Additionally, compared to a chemotherapy-free interval, maintenance therapy is associated with better PFS [162,163,164,165,166,167]. Several trials [162,163,164,165,166,167,168,169,170,171,172,173] including different maintenance strategies have been published (Table 4). Major guidelines recommend discussing maintenance therapy with patients and explaining side effect profiles of common options 5-FU/Leucovorin ± bevacizumab, cetixumab, or panitumumab [20,21,22].

#### 4.3.3. Second-Line and Subsequent Therapy

**Figure 6:**
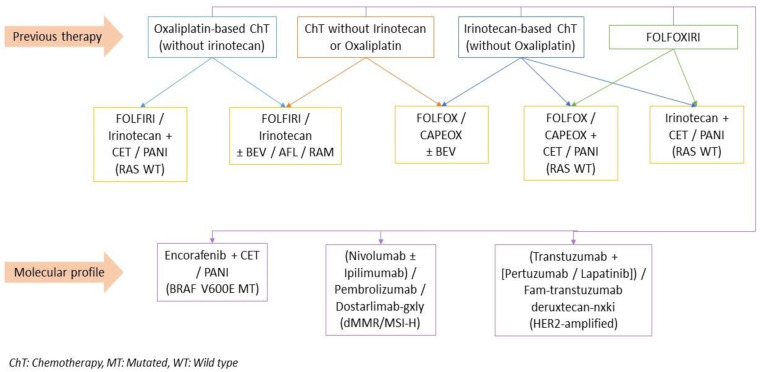
Second-line Systemic Treatment for Stage IV CRC.

Failure of systemic therapy is defined by the progression of metastatic disease. Second-line therapy describes systemic therapy options available after the failure of the first-line chemotherapy. Second-line therapy is tailored according to previous therapies (Figure 6). In general, patients who receive oxaliplatin-based chemotherapy upfront should be treated with irinotecan-based chemotherapy and vice versa [20,21,22]. The addition of biologics based on molecular profiling should be considered, given their association with increased OS [174,175,176]. Aflibercept and ramucirumab (in combination with FOLFIRI) can be considered as alternatives to bevacizumab for patients treated with oxaliplatin previously [175,176].

After progression on second-line therapy, patients with RAS/BRAF wild-type disease should receive an EGFR inhibitor combined with irinotecan. Alternatively, if they have HER2 mutation, transtuzumab can be preferred. If patients have BRAF V600E mutation, the encorafenib-cetuximab regimen should be considered [20,22]. Patients with RAS mutation who progressed under second-line therapy are considered to have the refractory disease [16]. Treatment with regorafenib or trifluridine and tipiracil is recommended for patients with refractory disease [16,20,22] as they may provide survival benefits compared to placebo [177,178].

## 5. Surveillance

The goal of surveillance in mCRC is to identify potentially resectable mCRC recurrences or new metachronous mCRC lesions. Surveillance protocols generally include follow-up visits with a clinical exam, tumor markers, imaging, and endoscopy. Generally, these follow-up visits occur up to 5 years after treatment, with more frequent surveillance in the first 2–3 years. This surveillance period is supported by evidence showing that 80–85% of CRC recurrence happens in the first 3 years, and less than 5% recur after 5 years [23,179,180]. Surveillance of mCRC is further justified by evidence showing surgical resection of recurrent mCRC can provide survival benefits. Butte et al. reported that up to 25% of patients that had resection of their mCRC recurrence were disease free at 36 months [181]. Therefore, surveillance can also help identify patients that are potentially curable of recurrent mCRC. It is important to note that evidence-based surveillance recommendations for mCRC are limited in the literature and the available recommendations are many times extrapolated from stage III CRC recommendations [182].

NCCN and ESMO have similar surveillance recommendations for history and physical (H&P) examination and CEA. Per NCCN, an H&P and CEA are recommended every 3 to 6 months (every 3 months per ESMO) for the first 2 years and then every 6 months for a total of 5 years. The ASCRS released guidelines in 2021, recommending an H&P and CEA every 3 to 12 months for the first 2 years and then every 6 to 12 months for the next 3 years. CEA surveillance routines are supported by the RCT, CEAwatch, which showed a significantly higher proportion of recurrence detected by CEA [183].

Most mCRC surveillance protocols recommend CT as the imaging modality of choice due to its ability to detect liver metastasis and resectable lung lesions [184]. For both colon and rectal cancer, NCCN and ESMO recommend a contrast-enhanced CT chest, abdomen, and pelvis every 3 to 6 months (3 months per ESMO) in the first 2 years and then every 6 to 12 months (6 months per ESMO) for up to a total of 5 years [20,21,22]. ASCRS has a less intense interval, recommending twice in 5 years or up to annually for 5 years in those with previously resected mCRC [185]. The variability in imaging frequency can likely be explained by a study from 2013, which showed that a higher frequency of imaging within 5 years did not result in increased survival in previously resected liver mCRC [186].

NCCN and ASCRS guidelines recommend colonoscopy 1 year after treatment, repeated in 3 years, and then every 5 years thereafter. For patients that did not have a preoperative colonoscopy due to an obstructing lesion, NCCN recommends a colonoscopy 3 to 6 months after surgery (1–6 months per ASCRS). Rectal cancer patients have additional surveillance depending on their treatment choice. For patients with transanal excision, NCCN recommends a rectal MRI or EUS every 3 to 6 months for 2 years, then every 6 months for a total of 5 years. ASCRS recommends a proctoscopy, possible rectal EUS, every 6 to 12 months for a total of 3 to 5 years in patients who underwent resection with anastomosis.

Other adjunct surveillance methods are being explored; however, no current evidence supports the addition of these to recent guidelines. PET/CT scans for routine surveillance are not recommended as they have not been shown to decrease mortality or detect resectable recurrence to justify the increased cost [187]. Additionally, there is insufficient evidence at the moment to recommend circulating DNA (ctDNA) as a surveillance method.

## 6. Future Direction of Stage IV Management

Although ctDNA does not have a role in the management of mCRC according to current guidelines, a recent large, prospective observational study including stage IV CRC patients demonstrated that postsurgical ctDNA at 4 weeks was a significant prognostic biomarker of recurrence. Kotani et al. [188] believe that ctDNA may be incorporated into staging criteria and predict adjuvant chemotherapy benefits in the future; however, randomized trials are lacking. Cancer stem cells are also being explored as a target of mCRC treatment due to their role in tumor growth, therapy resistance, metastasis, and relapse. However, no current clinical trials targeting CRC stem cells have been able to show relevant clinical activity in CRC treatment. Several trials were discontinued due to high toxicity and lack of anticancer activity [189]. Additionally, cancer stem cell biomarkers, like STAT3, are being explored for prognosis and patient-specific surveillance markers [190]. Tumor-driven changes have been observed in metabolic pathways such as glycolysis, lipid metabolism, and gut microbiota are also being explored as novel CRC treatment targets, but like cancer stem cells, pre-clinical research has not translated to promising clinical utility just yet [191].

## 7. Conclusions

CRC is the third most common cancer and the second cause of cancer-related mortality worldwide. Approximately 22% of patients with CRC have metastases at initial diagnosis. Management of mCRC is challenging, due to variable tumor extent and molecular characteristics. Unlike other stage IV cancers, surgical resection of metastases with curative intent is strongly recommended. Complete resection of liver, lung, and peritoneal metastases is associated with better disease control and survival. With the advances in systemic therapy, as well as a better understanding of tumor biology and the importance of molecular profiling, more patients can anticipate prolonged survival. Ultimately, a multidisciplinary evaluation of patients with mCRC is crucial to selecting the appropriate pathway.

## Figures and Tables

**Table 1:** Treatment of Colorectal Cancer with Liver/Lung Metastases.

Resectability of Metastases	Treatment Goal	Treatment Strategy
R0-resectable liver or lung metastases	Cure/No evidence of disease	R0 resection of the primary tumor and all metastases *Possible better long-term outcomes with perioperative ChT* Adjuvant therapy/Observation
Potentially resectable metastatic disease	Cure/No evidence of disease	Doublet/Triplet ChT ± targeted therapy R0 resection after conversion to resectable disease Adjuvant therapy/Observation
Unlikely to ever become resectable disease	Symptom control Prolonged survival Better QoL	Doublet/Triplet ChT ± targeted therapy *Surgery in the presence of (or high-risk for) primary tumor-related symptoms*

ChT: Chemotherapy, QoL: Quality of Life. R0 resection: Resection with histologically proven negative margins, in which no gross or microscopic tumor remains in the primary tumor bed. Conversion: Downstaging from unresectable to resectable disease.

**Table 2:** Oslo Score.

Maximum tumor diameter > 5.5 cm
CEA levels > 80 µg/L
Time from primary cancer surgery to liver transplantation < 2 years
Progression under chemotherapy

Each risk factor is equal to 1 point. An Oslo Score of 0–2 is associated with better overall survival.

**Table 3:** Selection Criteria for CRS ± HIPEC for CRC with Peritoneal Metastases.

ECOG performance status < 2
No major comorbidities (medically fit for surgery)
None-mild symptoms
Stable disease (no tumor progression) under chemotherapy
No extra-abdominal metastases *
Completeness of cytoreduction (CC score 0–1) possible
Peritoneal cancer index < 20
Patient’s motivation and informed consent

CRC: Colorectal Cancer, CRS: Cytoreductive Surgery, ECOG: Eastern Cooperative Oncology Group, HIPEC: Hyperthermic Intraperitoneal Chemotherapy. * Presence of resectable hepatic metastases is not a contraindication to CRS and HIPEC.

**Table 4:** Trials on Different Regimens for Maintenance Therapy.

Study (Trial Name)	Induction Chemotherapy	Maintenance Therapy	Outcomes	Results	Significance
Chibaudel 2009 [162] (GERCOR OPTIMOX2)	mFOLFOX7	FP vs. No treatment	PFS OS	8.6 vs. 6.6 months, HR 0.61 23.8 vs. 19.5 months, HR 0.88	***p* = 0.0017** *p* = NS
Hegewisch-Becker 2015 [163] (AIO 0207 #)	CAPOX/FOLFOX + BEVA	FP + BEVA vs. BEVA vs. No treatment	PFS OS Grade 3–4 AEs	6.3 vs. 4.6 vs. 3.5 months 20.2 vs. 21.9 vs. 23.1 30.4% vs. 24.3% vs. 13.3%	***p* < 0.0001** *p* = NS N/A *
Koeberle 2015 [164] (SAKK 41/06)	FP/FOLFOX/FOLFIRI + BEVA	BEVA vs. No treatment	PFS OS Grade 3–4 AEs	9.5 vs. 8.5 months, HR 0.75 25.4 vs. 23.8 months, HR 0.83 6.1% vs. 0.8%	***p* = 0.025** *p* = NS N/A *
Simkens 2015 [165] (CAIRO3)	CAPOX + BEVA	CAP/BEVA vs. No treatment	PFS OS Grade 3–4 AEs	8.5 vs. 4.1 months, HR 0.4 25.9 vs. 22.4 months, HR 0.83 60% vs. 34%	***p* < 0.0001** ***p* = 0.06** ***p* < 0.0001**
Luo 2016 [166]	CAPOX/FOLFOX	CAP vs. No treatment	PFS OS Grade 3–4 AEs	6.4 vs. 3.4 months, HR 0.54 25.6 vs. 23.3 months, HR 0.85 41.9% vs. 22.4%	***p* < 0.001** *p* = NS N/A *
Aparicio 2018 [167] (PRODIGE 9)	FOLFIRI + BEVA	BEVA vs. No treatment	PFS PFS rate (12 months) OS	9.2 vs. 8.9 months, HR 0.91 30.2% vs. 21% 21.7 vs. 22 months, HR 1.11	*p* = NS *p* = NS *p* = NS
Dìaz-Rubio 2012 [168] (MACRO TTD)	CAPOX + BEVA	BEVA vs. Continuation of ChT	PFS OS Grade 3–4 AEs	9.7 vs. 10.4 months, HR 1.10 20 vs. 23.2 months, HR 1.05 55% vs. 47%	*p* = NS *p* = NS N/A *
Yalcin 2013 [169] (Stop and Go)	CAPOX + BEVA	CAP + BEVA vs. Continuation of ChT	PFS OS Grade 3–4 AEs	11.0 vs. 8.3 months, HR 0.6 23.8 vs. 20.2 months 34.4% vs. 48.4%	***p* = 0.002** *p* = NS *p* = NS
Hagman 2016 [170] (Nordic ACT2)	CAPOX/FOLFOX or CAPIRI/FOLFIRI ± BEVA	BEVA vs. BEVA + ERLO (KRAS WT)	PFS PFS rate (3 months) OS Grade 3–4 AEs	3.6 vs. 5.7 months, HR 0.93 64.7% vs. 63.6% 30.7 vs. 20.6 months, HR 0.58 25.7% vs. 58.2%	*p* = NS *p* = NS *p* = 0.051 N/A *
		BEVA vs. CAP (KRAS MT)	PFS PFS rate (3 months) OS Grade 3–4 AEs	3.9 vs. 3.7 months, HR 1.19 75% vs. 66.7% 26.4 vs. 28.0 months, HR 1.57 20.6% vs. 15.2%	*p* = NS *p* = NS *p* = NS N/A *
Cremolini 2018 [171]	mFOLFOXIRI + CET	CET vs. BEVA (KRAS WT)	PFS OS Grade 3–4 AEs	13.3 vs. 10.8 months, HR 0.73 37.5 vs. 37 months, HR 0.98 25% vs. 8%	*p* = NS *p* = NS N/A *
Pietrantonio 2019 [172]	FOLFOX + PANI	FP + PANI vs. PANI	PFS rate (10 months) OS rate (18 months) Grade 3–4 AEs	59.9% vs. 49% 66.4% vs. 62.4% 42.4% vs. 20.3%	***p* = 0.01** *p* = NS N/A *
Modest 2021 [173] (PANAMA)	FOLFOX + PANI	FP + PANI vs. FP	PFS OS	8.8 vs. 5.7 months, HR 0.72 28.7 vs. 25.7 months, HR 0.84	***p* = 0.014** *p* = NS

BEVA: Bevacizumab, CAP: Capecitabine, CET: Cetuximab, ERLO: Erlotinib, FP: Fluoropyrimidine, PANI: Panitumumab. AE: Adverse events, ChT: Chemotherapy, HR: Hazard ratio, MT: Mutated, NS: Non-significant, OS: Overall survival, PFS: Progression-free survival, WT: Wild-type. * No information regarding significance level. # BEVA alone was non-inferior to FP + BEVA, whereas no treatment was not. Bold indicates that the difference between the results are statistically significant.

## Data Availability

No new data was created as this article was a literature review.
